# Rare earth doping: a strategy to enhance the catalytic activity of ZnFe_2_O_4_ in activating peroxymonosulfate for acetaminophen degradation

**DOI:** 10.1039/d5ra05044h

**Published:** 2025-08-12

**Authors:** Hangdao Qin, Lei Xiao, Junnan Hao, Yong Wang, Jiming Huang, Guo Yang, Bo Xing

**Affiliations:** a School of Material and Chemical Engineering, Tongren University Tongren 554300 China qinhangdao@126.com; b College of Chemical Engineering, Sichuan University of Science and Engineering Zigong 643000 China

## Abstract

In this paper, ZnFe_2_O_4_ was doped by two rare earth metals (La and Pr), and the as-prepared ZnLa_0.5_Fe_1.5_O_4_ and ZnPr_0.5_Fe_1.5_O_4_ were applied to activate PMS for acetaminophen degradation. ZnLa_0.5_Fe_1.5_O_4_ with the largest oxygen vacancy (O_V_) content showed the highest acetaminophen degradation efficacy. About 89.7% of acetaminophen was removed within 60 min in the ZnLa_0.5_Fe_1.5_O_4_/PMS system. Free radical quenching experiments and EPR tests confirmed that SO_4_˙^−^, ˙OH, O_2_˙^−^ and ^1^O_2_ were the dominant reactive oxygen species (ROS). The role of La/Pr doping was explored through a series of comparative studies. The results indicated that La doping enhanced the content of oxygen vacancies, accelerated the electron transfer in the system, and thus sharply improved the catalytic performance of ZnFe_2_O_4_. Furthermore, the reusability, universality and actual water environment adaptability of ZnLa_0.5_Fe_1.5_O_4_ were investigated.

## Abstract 2

La/Pr doping enhanced the content of oxygen vacancies, accelerated the electron transfer in the system, and thus sharply improved the catalytic performance of ZnFe_2_O_4_.

## Graphical abstract

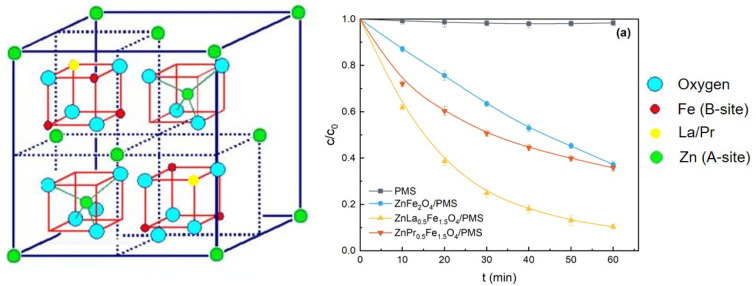

## 1. Introduction

Sulfate radical-based advanced oxidation processes (SR-AOPs) have been proved to effectively eliminate pharmaceuticals and personal care products (PPCPs) in water due to the generation of powerful reactive oxygen species (ROS) in the presence of catalysts.^1^ ZnFe_2_O_4_ is a bimetallic oxide with spinel structure (AB_2_O_4_), and bivalent Zn^2+^ occupies the A-site and trivalent Fe^3+^ occupies the B-site. Magnetic nanostructured ZnFe_2_O_4_ is a highly efficient and environmentally friendly catalyst in activating peroxymonosulfate (PMS) for the degradation of organic pollutants.^2,3^

To further improve the catalytic performance of ZnFe_2_O_4_, metal cation doping has been proved to be a feasible method.^4–6^ Mn-doped ZnFe_2_O_4_ (Mn_0.6_Zn_0.4_Fe_2_O_4_) was prepared using spent Zn-Mn alkaline batteries, and its catalytic performance was higher than that of ZnFe_2_O_4_ and MnFe_2_O_4_ in activating PMS for bisphenol A degradation.^4^ In the previous work of the authors, Ni-doped ZnFe_2_O_4_ (Ni_0.5_Zn_0.5_Fe_2_O_4_) was synthesized and applied to activate PMS for the decay of cefotaxime sodium.^5^ The results indicated that the synergistic effect between Ni^2+^ and Zn^2+^ accelerated the Fe^3+^/Fe^2+^ cycle, and thus promoted the generation of ROS in the system. In another report, Cu substituted ZnFe_2_O_4_ was successfully prepared and used for PMS activation to degrade ciprofloxacin.^6^ The excellent catalytic activity was due to the generated large amount of oxygen vacancies after Cu substitution. Oxygen vacancies were also the main active sites for PMS activation to produce ROS. It could be concluded that doping could not only accelerate the electron transfer in the system by the synergistic effect between metals, but also regulate the generation of oxygen vacancies in ZnFe_2_O_4_, which was beneficial to promote the production of ROS in the catalytic system. Oxygen vacancies were curial.

However, all the above reports were focused on A-site doping, the effect of B-site doping on the catalytic performance of ZnFe_2_O_4_ was rarely investigated. In this study, trivalent rare earth ions (La^3+^ and Pr^3+^) were used to partly substitute Fe^3+^ in B-site. The as-prepared catalysts were applied to activate PMS for the degradation of acetaminophen which was a widely used antipyretic and analgesic pharmaceutical.^7^ The effect of rare earth cation doping on the microstructure, oxygen vacancies and catalytic activity was studied based on the catalyst characterization and activity evaluation. Furthermore, the produced ROS were determinated and the role of La/Pr doping was investigated based on a series of comparative studies including X-ray photoelectron spectra (XPS) analysis, electron paramagnetic resonance (EPR) tests and electrochemical analysis. Finally, the reusability, universality and actual water environment adaptability of the catalyst were investigated to evaluate the potential of practical applications of the catalyst.

## 2. Experimental

### 2.1. Catalyst preparation and characterization

The sourcing and purity information of the used chemicals in this study are mentioned in Text S1 in the SI.

La/Pr-doped ZnFe_2_O_4_ was prepared by sol–gel self-combustion method. The detailed synthesis process was described in the previous study.^8^ Synthesis schematic depiction of ZnLa_0.5_Fe_1.5_O_4_/ZnPr_0.5_Fe_1.5_O_4_ catalyst has been added in Fig. S1 in the SI. Took ZnLa_0.5_Fe_1.5_O_4_ as an example. Analytical grade Zn(NO_3_)_2_·9H_2_O, Fe(NO_3_)_3_·9H_2_O and La(NO_3_)_3_·6H_2_O were accurately weighed in stoichiometric proportions, and then dissolved in distilled water. Citric acid was added into each sample of metal nitrates in 1 : 1 molar ratio of citric acid to metal ions. The above solutions were heated for 5 h at 70 °C through gel phase transformation. The dry gel was obtained after aging and drying, and then ground into powder and heat-treated at 500 °C for 2 h in a muffle furnace with the heating rate of 10 °C min^−1^. La(NO_3_)_3_·6H_2_O was replaced with Pr(NO_3_)_3_·5H_2_O when preparing ZnPr_0.5_Fe_1.5_O_4_. Undoped ZnFe_2_O_4_, as a control, was synthesized using the same procedure without adding dopant. The detailed information including instruments and methods used in catalyst characterization was shown in Text S2 in the SI.

### 2.2. Degradation experiments and analytical methods

The degradation experiments were carried out in a 250 mL conical flask, which was placed in a constant temperature air bath shaker. The temperature and the rotate speed were set at 25 °C and 300 rpm, respectively. In each experiment, 100 mg of solid catalyst was introduced into 100 mL of 20 mg L^−1^ acetaminophen solution. Then 100 mg of PMS was introduced into the system to initial the oxidation reaction. Samples were extracted at specific time intervals and immediately filtered through a 0.45 μm membrane for further analysis. The detailed experimental procedure for the recycle study was shown in Text S3 in the SI. The analytical methods were described in detail in Text S4 in the SI. The details of operational approach of electrochemical impedance spectroscopy (EIS) and open-circuit potential (OCP) were presented in Text S5 in the SI.

## 3. Results and discussion

### 3.1. Characterization

**Fig. 1:**
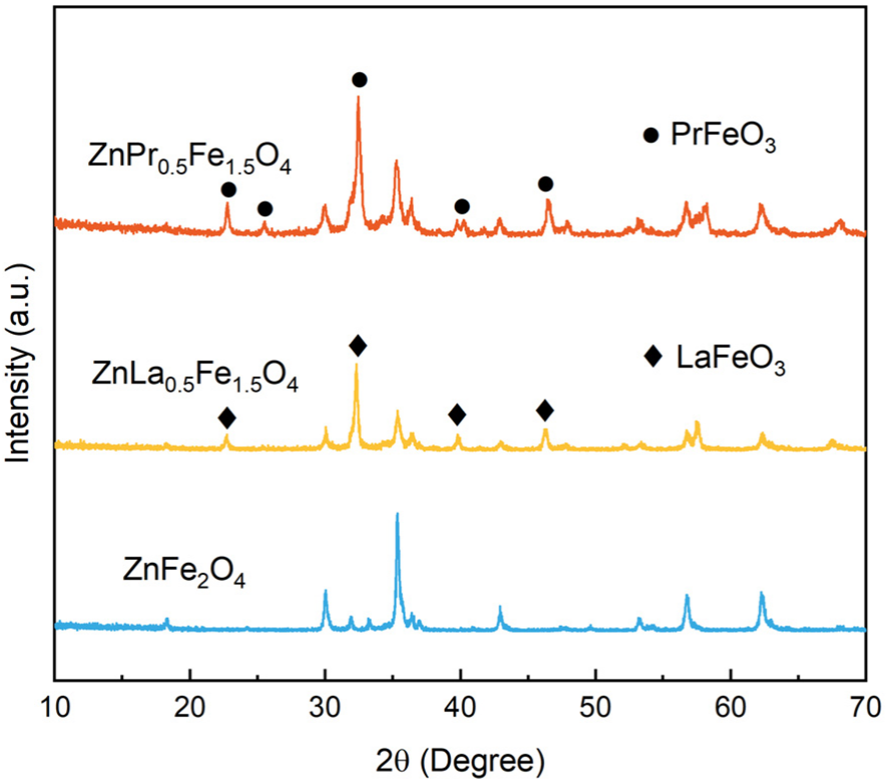
XRD patterns of ZnFe_2_O_4_, ZnLa_0.5_Fe_1.5_O_4_ and ZnPr_0.5_Fe_1.5_O_4_.

The X-ray diffraction (XRD) patterns of the as-prepared catalysts are shown in Fig. 1. All of these catalysts exhibited a pure cubic crystal structure of franklinite (JCPDS No. 89-4926). Some new peaks appeared after doping, which corresponded to the crystal planes of LaFeO_3_ (JCPDS No. 88-0641) and PrFeO_3_ (JCPDS No. 47-0065) with perovskite structure.^9,10^ These results indicated that a composite of spinel ZnFe_2_O_4_ and perovskite LaFeO_3_/PrFeO_3_ was formed when parts of framework Fe were substituted by La/Pr.

**Fig. 2:**
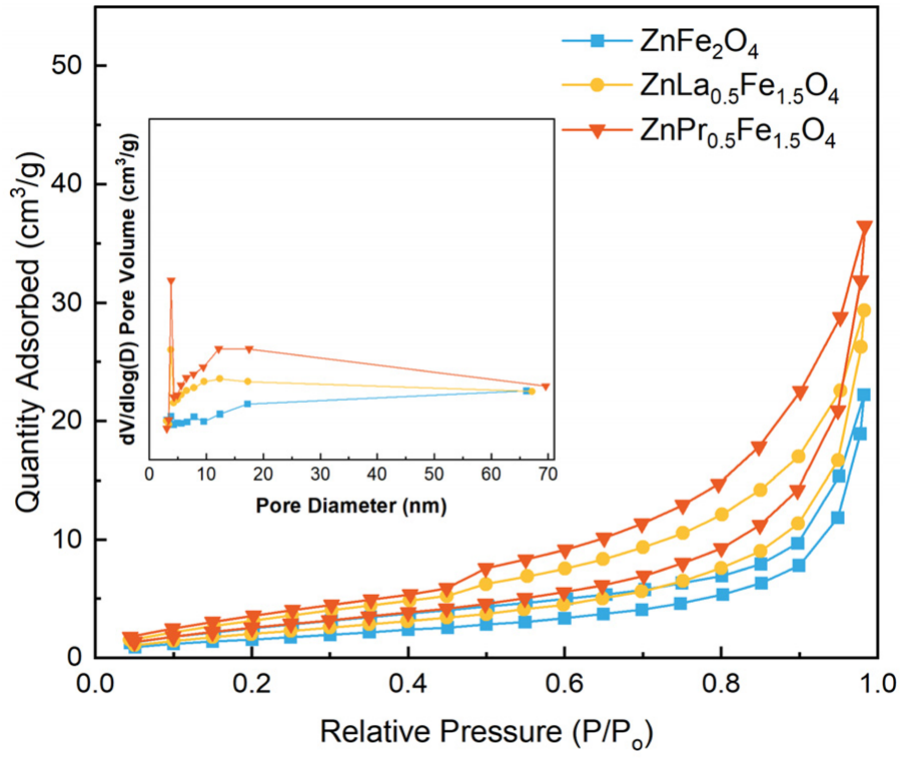
N_2_ adsorption–desorption isotherms and pore size distribution curves of ZnFe_2_O_4_, ZnLa_0.5_Fe_1.5_O_4_ and ZnPr_0.5_Fe_1.5_O_4_.

The N_2_ adsorption–desorption isotherms and pore size distribution curves of catalysts are presented in Fig. 2. All the isotherms possessed the type IV curve with an H3 type hysteresis loop, which suggested a mesoporous structure of the four catalysts. Moreover, the pore size distribution curves indicated that the pore size basically concentrated in the range of 1–20 nm. The BET surface area was calculated from the Brunauer–Emmett–Teller (BET) method, and the total pore volume was determined by the near saturation uptake (*P*/*P*_0_ = 0.99). The average pore size was calculated using Barrett–Joyner–Halenda (BJH) method. As seen from Table 1 that rare earth doping enhanced the surface area and pore volume, and ZnPr_0.5_Fe_1.5_O_4_ showed higher BET surface area (20 m^2^ g^−1^) and pore volume (0.058 cm^3^ g^−1^).

**Table 1:** The BET surface area, pore volume and average pore size of catalysts

Catalysts	BET surface area (m^2^ g^−1^)	Pore volume (cm^3^ g^−1^)	Average pore size (nm)
ZnFe_2_O_4_	8	0.033	3.07
ZnLa_0.5_Fe_1.5_O_4_	15	0.046	3.82
ZnPr_0.5_Fe_1.5_O_4_	20	0.058	3.82

### 3.2. Removal of acetaminophen in different systems

**Fig. 3:**
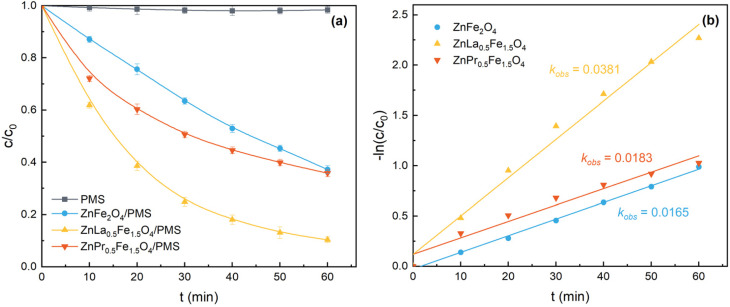
(a) Acetaminophen degradation in different systems; (b) acetaminophen degradation kinetics. Reaction conditions: [acetaminophen]_0_ = 20 mg L^−1^, [PMS]_0_ = 1.0 g L^−1^, [catalyst]_0_ = 1.0 g L^−1^, unadjusted pH = 6.98, *T* = 25 °C.

The adsorption of acetaminophen by different catalysts was investigated (Fig. S2), and the results indicated that adsorption could hardly remove acetaminophen. As shown in Fig. 3a, single PMS had low degradation ability for acetaminophen, since only 1.2% of acetaminophen was removed within 60 min. The degradation rate of acetaminophen was 62.8% in ZnFe_2_O_4_/PMS system. La/Pr doping improved the catalytic performance, and ZnLa_0.5_Fe_1.5_O_4_/PMS system (89.7%) showed higher acetaminophen degradation efficiency than that of ZnPr_0.5_Fe_1.5_O_4_/PMS system (64.2%). Moreover, according to Fig. 3b, the degradation of acetaminophen could be well fitted by the pseudo-first-order kinetic. The *k*_obs_ was calculated to be 0.0165, 0.0183 and 0.0381 min^−1^ for ZnFe_2_O_4_, ZnLa_0.5_Fe_1.5_O_4_ and ZnPr_0.5_Fe_1.5_O_4_, respectively. The catalytic performances followed the order of ZnFe_2_O_4_ < ZnPr_0.5_Fe_1.5_O_4_ < ZnLa_0.5_Fe_1.5_O_4_.

Besides, the ZnLa_0.5_Fe_1.5_O_4_ catalyst obtained in the present study was compared with other catalysts used in acetaminophen degradation (Table 2). Although all the catalysts were used in AOPs for the degradation of acetaminophen, the oxidizing reagents and reaction conditions were different, and thus a true quantity comparison was impossible.

**Table 2:** The ZnLa_0.5_Fe_1.5_O_4_ catalyst compared with other catalysts used in acetaminophen degradation

Catalyst	Oxidizing reagent	Removal	References
ZnLa_0.5_Fe_1.5_O_4_	PMS	89.7%	This study
Co/EMR	H_2_O_2_	63.8%	Qin *et al.*^7^
Fe-g-C_3_N_4_	H_2_O_2_	98.1%	Mu *et al.*^11^
C/TiO_2_	PDS	100%	Dang *et al.*^12^
FeMo@NCN	PMS	72.0%	Huang *et al.*^13^
FeCe_0.05_/BC	PMS	99.9%	Du *et al.*^14^

### 3.3. The role of La/Pr doping

#### 3.3.1 Influence of oxygen vacancies

It was observed that there was a lack of linear correlation between catalytic performance and BET surface area (Table 1). Therefore, in order to explore the role of La/Pr doping, a series of comparative studies including XPS analysis, EPR tests and electrochemical analysis were conducted.

**Fig. 4:**
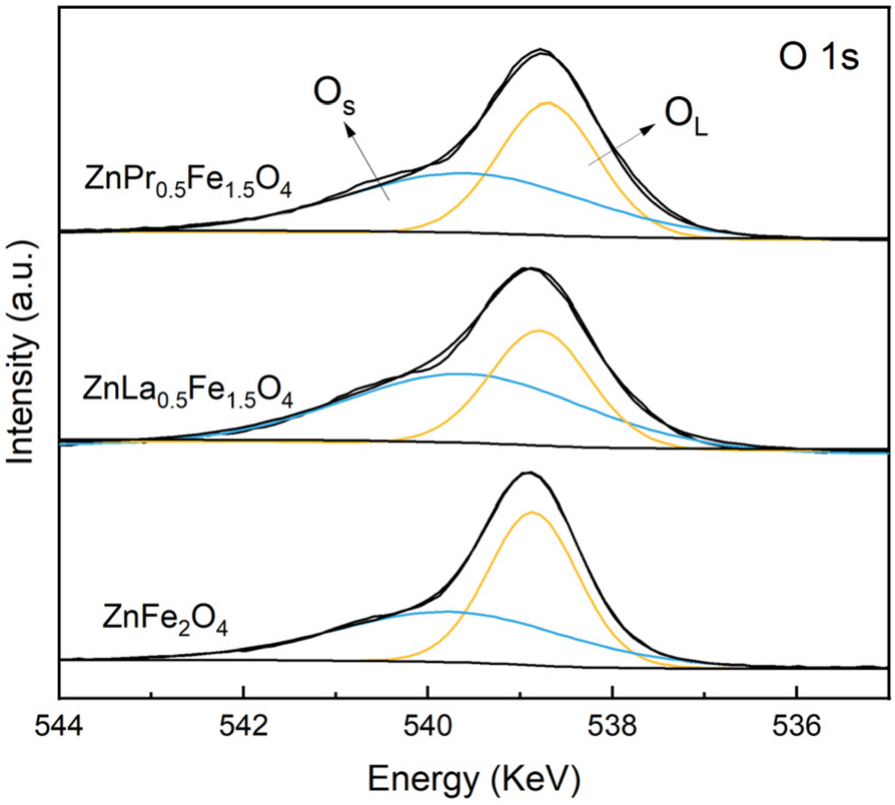
O 1s XPS spectra of ZnFe_2_O_4_, ZnLa_0.5_Fe_1.5_O_4_ and ZnPr_0.5_Fe_1.5_O_4_.

Firstly, XPS analysis and EPR tests were conducted to clarify the relationship between the doping elements and oxygen vacancies in the catalysts. The XPS survey spectra of ZnFe_2_O_4_, ZnLa_0.5_Fe_1.5_O_4_ and ZnPr_0.5_Fe_1.5_O_4_ were presented in Fig. S3 in the SI. ZnLa_0.5_Fe_1.5_O_4_ consisted of Zn, Fe, La and O elements and ZnPr_0.5_Fe_1.5_O_4_ consisted of Zn, Fe, Pr and O elements, which further confirmed that La/Pr was successfully doped onto ZnFe_2_O_4_. The fine-scanned XPS spectra of O 1 s were shown in Fig. 4. The peaks at 538.6 eV and 539.5 eV were assigned to lattice oxygen (O_L_) and adsorbed oxygen or surface oxygen (O_S_), respectively. The ratio of O_S_/O_L_ was closely related to oxygen vacancies, and the high ratio signified more oxygen vacancies over the catalyst surface.^15^ The calculated ratios were 0.85, 1.54 and 1.14 for ZnFe_2_O_4_, ZnLa_0.5_Fe_1.5_O_4_ and ZnPr_0.5_Fe_1.5_O_4_, respectively. ZnLa_0.5_Fe_1.5_O_4_ possessed the most oxygen vacancies, while the content of oxygen vacancies in ZnFe_2_O_4_ was smallest.

**Fig. 5:**
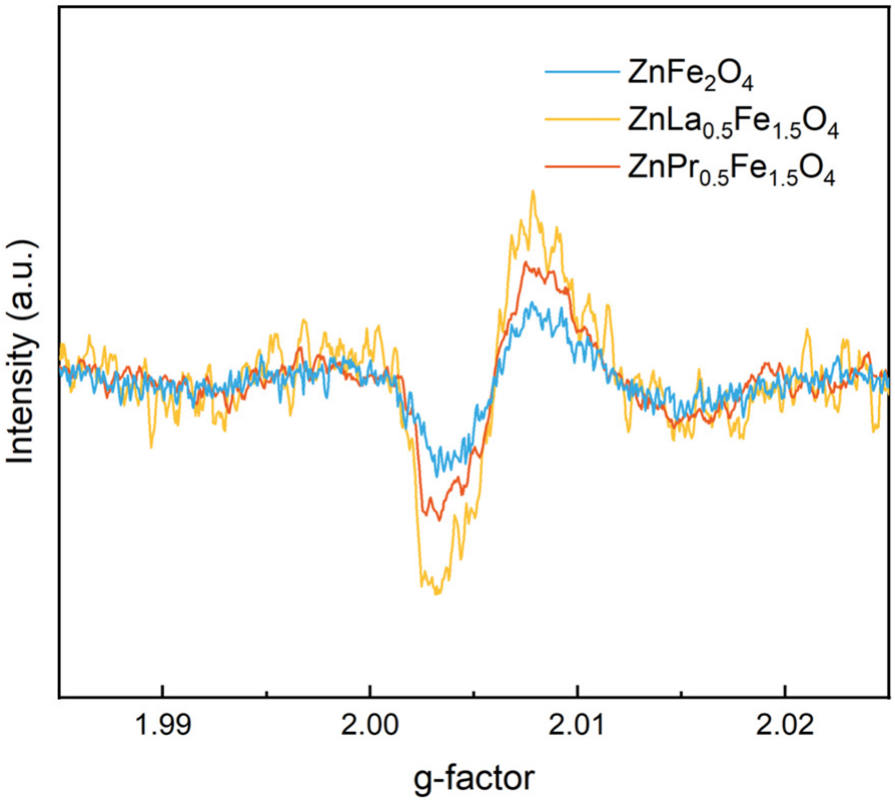
EPR spectra of ZnFe_2_O_4_, ZnLa_0.5_Fe_1.5_O_4_ and ZnPr_0.5_Fe_1.5_O_4_.

Moreover, as presented in Fig. 5, the intensity of the EPR signals followed the order of ZnFe_2_O_4_ < ZnPr_0.5_Fe_1.5_O_4_ < ZnLa_0.5_Fe_1.5_O_4_. Based on the results of XPS analysis and EPR tests, it could be concluded that as same as A-site doping, B-site doping with rare earth also could tune the oxygen vacancies in ZnFe_2_O_4_ catalyst, and the oxygen vacancies were increased by the incorporation of rare earth into the lattice. It was important that the catalytic activity was increased with the concentration of oxygen vacancies in the catalysts, which indicated oxygen vacancies were the active sites for the activation of PMS.^16^

#### 3.3.2 ROS determination

**Fig. 6:**
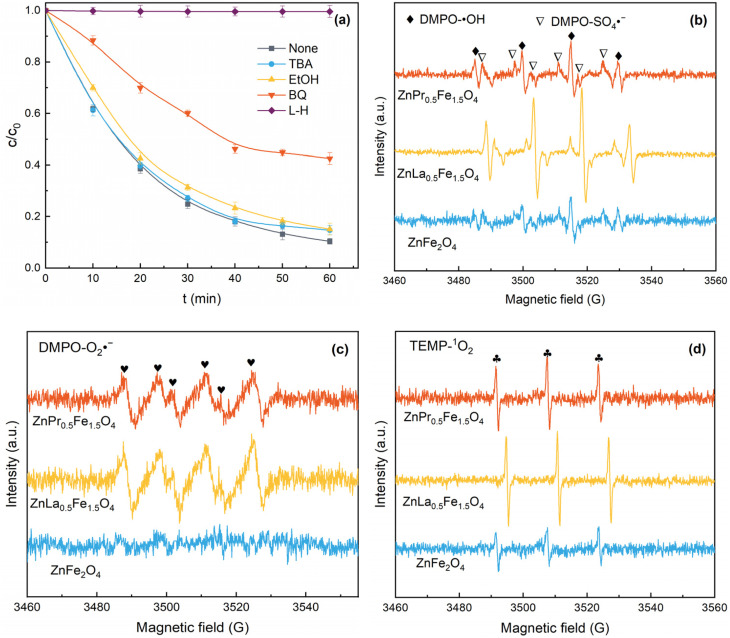
(a) Quenching experiments; EPR spectra of ˙OH and SO_4_˙^−^ (b), O_2_˙^−^ (c), and ^1^O_2_ (d). Reaction conditions: [acetaminophen]_0_ = 20 mg L^−1^, [PMS]_0_ = 1.0 g L^−1^, [catalyst]_0_ = 1.0 g L^−1^, [TBA] = [EtOH] = [BQ] = [l-H] = 10 mM, unadjusted pH = 6.98, *T* = 25 °C.

It was known that PMS could be activated to produce ROS to oxidize organic pollutants. Free radicals quenching experiments were performed to determinate the generated ROS in ZnLa_0.5_Fe_1.5_O_4_/PMS system, and the results were illustrated in Fig. 6a. The degradation of acetaminophen was almost completely inhibited when l-Histidine (l-H) was introduced. The addition of benzoquinone (BQ) apparently prevented the removal of acetaminophen. However, after adding *tert*-butanol (TBA) or ethanol (EtOH) into the system, the acetaminophe degradation rate declined slightly. These results suggested O_2_˙^−^ and ^1^O_2_ contributed more than SO_4_˙^−^ and ˙OH for the decay of acetaminophe.

Moreover, EPR measurements were used to further confirm the ROS generated during the catalytic process. In Fig. 6b, 5,5-dimethyl-1-pyrroline-*N*-oxide (DMPO) was used as a radical trapping agent for SO_4_˙^−^ and ˙OH, and DMPO-SO_4_˙^−^ and DMPO-˙OH signals were observed, which indicated both SO_4_˙^−^ and ˙OH were generated in ZnLa_0.5_Fe_1.5_O_4_/PMS system. As shown in Fig. 6c, DMPO was used as a radical trapping agent for O_2_˙^−^, and the characteristic signal for DMPO-O_2_˙^−^ adducts confirmed the generation of O_2_˙^−^ in ZnLa_0.5_Fe_1.5_O_4_/PMS system. As exhibited in Fig. 6d, 2,2,6,6-tetramethyl-4-piperidone (TEMP) was employed as a radical trapping agent for ^1^O_2_, and the typical 1 : 1 : 1 triplet signals of TEMP-^1^O_2_ were detected, which further demonstrated the existence of ^1^O_2_ in ZnLa_0.5_Fe_1.5_O_4_/PMS system. Besides, the signal intensity of ZnLa_0.5_Fe_1.5_O_4_ was higher than that of ZnFe_2_O_4_ and ZnPr_0.5_Fe_1.5_O_4_, which was the reason for the highest catalytic performance of ZnLa_0.5_Fe_1.5_O_4_. La/Pr doping enhanced the content of oxygen vacancies, and thus accelerated the production of ROS, which was ascribed to the fact that oxygen vacancies could facilitate the adsorption of PMS on catalyst surface.^17^

#### 3.3.3 Confirmation of direct electron transfer pathway

**Fig. 7:**
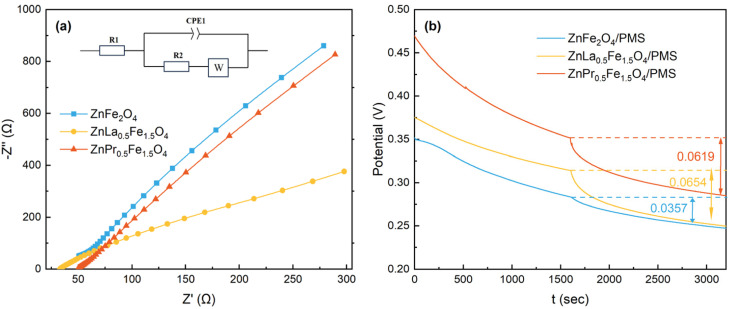
EIS curves (a) and OCP curves (b) of different electrodes.

Except for oxidizing by ROS, the electron transfer pathway might be involved in the degradation of organic contaminations. On the other hand, the oxygen vacancies could facilitate the interfacial electron transfer of the catalyst,^18^ so the electrochemical impedance spectroscopy (EIS) was used to determinate the charge transfer resistances for the three catalysts. As shown in Fig. 7a, the charge transfer resistance (*R*_ct_) obtained from equivalent circuit fitting was 7368, 824.1 and 2325 Ω for ZnFe_2_O_4_, ZnLa_0.5_Fe_1.5_O_4_ and ZnPr_0.5_Fe_1.5_O_4_, respectively. The results demonstrated that La/Pr doping elevated electronic conductivity and charge transfer efficiency. Therefore, La/Pr doping was able to enhance the reaction kinetic performance of the catalytic system by lowing the charge transfer barrier. In addition, the electron transformation ability followed the order of ZnLa_0.5_Fe_1.5_O_4_ > ZnPr_0.5_Fe_1.5_O_4_ > ZnFe_2_O_4_, which was the same as the order of the content of oxygen vacancies in the three catalysts.

Moreover, the charge transfer among PMS, catalyst and acetaminophe was also explored through the open-circuit potential (OCP) curves. As illustrated in Fig. 7b, after the addition of acetaminophe into the different catalytic systems, an obvious decline of potential was observed, which was ascribed to the donation of electrons by acetaminophe. The potential dropped due to the decomposition of PMS and the oxidation of metal ions.^16^ The potential difference was 0.0367 V, 0.0654 V and 0.0619 V in ZnFe_2_O_4_/PMS system, ZnLa_0.5_Fe_1.5_O_4_/PMS system and ZnPr_0.5_Fe_1.5_O_4_/PMS system, respectively. The potential difference in the ZnLa_0.5_Fe_1.5_O_4_/PMS system was the largest, which was contributed to the highest degradation efficiency of acetaminophe.

Overall, the oxidation of ROS and the direct electron transfer were responsible for the degradation of acetaminophe. More importantly, based on the above comparative studies, it could be concluded that La/Pr doping enhanced the content of oxygen vacancies in ZnFe_2_O_4_, and the enriched oxygen vacancies promoted the generation of ROS and accelerated the electron transfer in the system, and thus improved the degradation rate of acetaminophe.

### 3.4. Practical application potential assessment

**Fig. 8:**
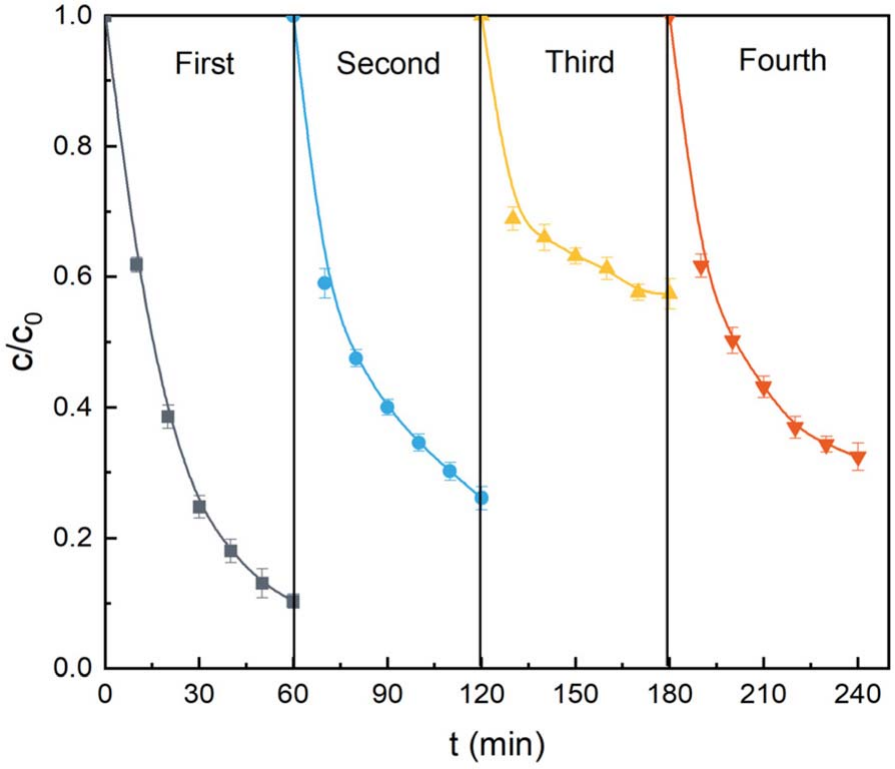
The reusability of ZnLa_0.5_Fe_1.5_O_4_ for acetaminophe degradation. Reaction conditions: [acetaminophen]_0_ = 20 mg L^−1^, [PMS]_0_ = 1.0 g L^−1^, [catalyst]_0_ = 1.0 g L^−1^, unadjusted pH = 6.98, *T* = 25 °C.

In order to evaluate the potential of practical applications of ZnLa_0.5_Fe_1.5_O_4_, its reusability, actual water environment adaptability and universality were investigated. Firstly, the reusability of ZnLa_0.5_Fe_1.5_O_4_ was evaluated in the four continuous cycling tests. No regeneration method was carried out in the first three cycles, and the used ZnLa_0.5_Fe_1.5_O_4_ was heat-treated 2 h at 500 °C before the fourth cycle. As presented in Fig. 8, the degradation efficiency towards acetaminophe was 89.7%, 73.9% and 42.6% in the first, second and third cycle, respectively. The decrease of catalytic performance could be caused by the occupation of active sites by intermediate products and the unavoidable loss of catalyst in the recovery process.^19,20^ The acetaminophe degradation efficiency was 67.5% in the fourth cycle, which indicated that the heat-treatment could lead to a partial recovery of the catalytic performance of ZnLa_0.5_Fe_1.5_O_4_.

**Fig. 9:**
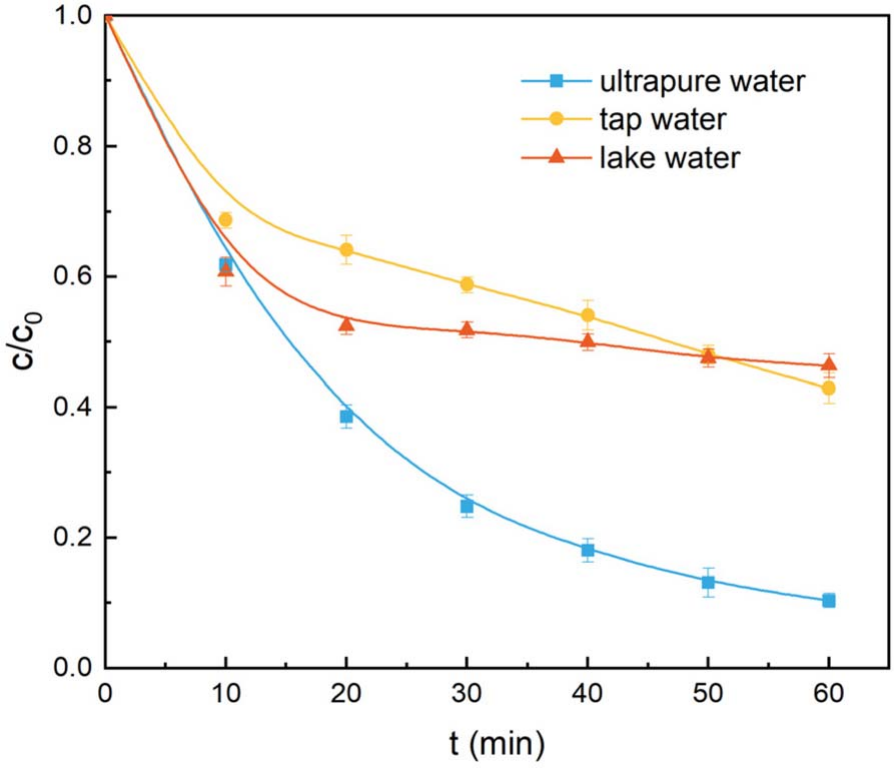
The degradation of acetaminophen in different waterbodies. Reaction conditions: [acetaminophen]_0_ = 20 mg L^−1^, [PMS]_0_ = 1.0 g L^−1^, [catalyst]_0_ = 1.0 g L^−1^, unadjusted pH = 6.98, *T* = 25 °C.

To examine whether ZnLa_0.5_Fe_1.5_O_4_ could be satisfactorily applied to actual water environment, the acetaminophe degradation in tap water and lake water (collected from the Mingde Lake in Tongren University, Tongren, China) was also conducted. As shown in Fig. 9, the removal efficiency of acetaminophe followed the order of lake water < tap water < ultrapure water. The decrease of the acetaminophe removal efficiency could be due to the existence of Cl^−^ in the tap water and the existence of humic substances and Cl^−^ in and lake water.^21,22^

**Fig. 10:**
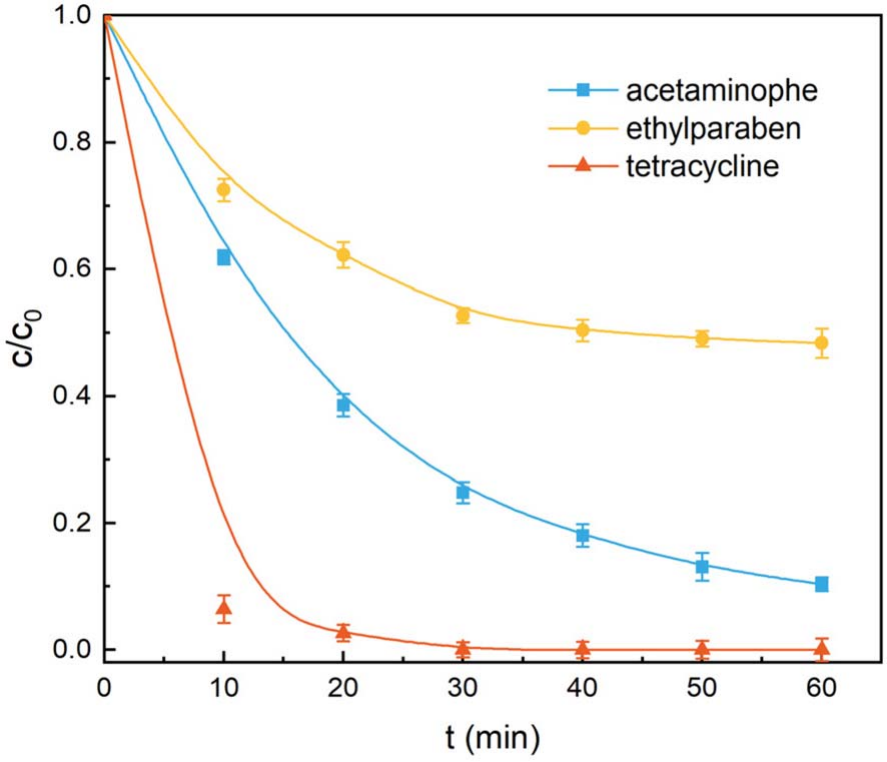
Removal of various PPCPs using ZnLa_0.5_Fe_1.5_O_4_/PMS system. Reaction conditions: [acetaminophen]_0_ = [ethylparaben]_0_ = [tetracycline]_0_ = 20 mg L^−1^, [PMS]_0_ = 1.0 g L^−1^, [catalyst]_0_ = 1.0 g L^−1^, unadjusted pH, *T* = 25 °C.

The universality of ZnLa_0.5_Fe_1.5_O_4_ catalyst was evaluated through the degradation of different PPCPs in ZnLa_0.5_Fe_1.5_O_4_/PMS system. It could be seen from Fig. 10 that tetracycline could be completely degraded within 30 min. Although the removal rate of ethylparaben was 51.6%, it could be further improved under the optimal reaction conditions. These results indicated ZnLa_0.5_Fe_1.5_O_4_/PMS system was effective and possessed potential application in the degradation of other PPCPs.

### 3.5. Determination of intermediates and toxicity estimation

**Fig. 11:**
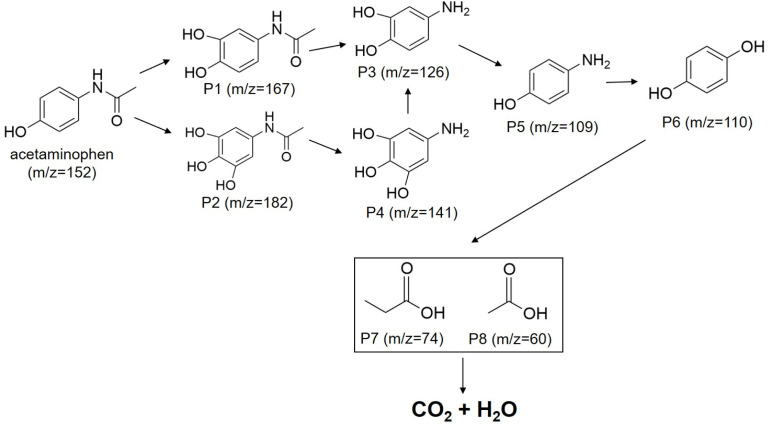
Poosible degradation pathways of acetaminophen in ZnLa_0.5_Fe_1.5_O_4_/PMS system.

The possible degradation pathway of acetaminophen in ZnLa_0.5_Fe_1.5_O_4_/PMS system was proposed based on the liquid chromatography-mass spectrography (LC-MS) analysis. As shown in Fig. 11, the acetaminophen (*m*/*z* = 152) was directly attacked by ˙OH, resulting in the formation of P1 (*m*/*z* = 167) and P2 (*m*/*z* = 182).^11^ Then, O_2_˙^−^ attacked the C–N bond of P1 and P2, and the acetyl group was removed to form P3 (*m*/*z* = 126) and P4 (*m*/*z* = 141), respectively.^12^ P3 and P4 were converted to P5 (*m*/*z* = 109) under sustained ROS attack, and the amino group of P5 was oxidized to form P6 (*m*/*z* = 110). Subsequently, P6 was ring-opened to produce P7 (*m*/*z* = 74) and P8 (*m*/*z* = 60). Finally, these small carboxylic acids were mineralized to form CO_2_ and H_2_O.

The toxicities of acetaminophen and the eight intermediates were evaluated by Toxicity Estimation Software Tool (TEST). As presnted in Fig. S4 in the SI, the original BHA exhibited “developmental toxicant”. After degradation in ZnLa_0.5_Fe_1.5_O_4_/PMS system, the developmental toxicity values of P3, P4, P5, P6, P7 and P8 were smaller than that of acetaminophen. Theses results indicated that the overall toxicity of acetaminophen could be weakened by using ZnLa_0.5_Fe_1.5_O_4_/PMS system.

## 4. Conclusion

In conclusion, rare earth ions were used to partly substitute Fe^3+^ in B-site, and La/Pr-doped ZnFe_2_O_4_ was successfully prepared and used to activating PMS for the degradation of acetaminophen. The catalytic performance of ZnFe_2_O_4_, ZnLa_0.5_Fe_1.5_O_4_ and ZnPr_0.5_Fe_1.5_O_4_ nanoparticles presented a positive correlation with the content of their oxygen vacancies. ZnLa_0.5_Fe_1.5_O_4_ with the highest amount of oxygen vacancies showed the highest catalytic activity, allowing an 89.7% removal efficiency of acetaminophen within 60 min. Both the characterizations and electrochemical analysis indicated the enhanced catalytic activity could be ascribed to the promotion of generated ROS and the acceleration of the electron transfer in the system. Moreover, the reusability experiments indicated that the degradation efficiency of acetaminophe decreased from 89.7% to 42.6% in the third cycle, and the heat-treatment could lead to a partial recovery of the catalytic performance of ZnLa_0.5_Fe_1.5_O_4_.

This work demonstrated that tuning the oxygen vacancies induced by La/Pr doping was a feasible strategy for enhancing the catalytic activity of ZnFe_2_O_4_, which provided a theoretical basis for the improvement of catalytic performance by doping of metals. The ZnLa_0.5_Fe_1.5_O_4_/PMS system showed enormous potential for treating PPCPs in water. However, further studies, such as the optimum doping content and the performance of ZnLa_0.5_Fe_1.5_O_4_ in the treatment of real wastewater remain to be investigated.

## Conflicts of interest

The authors declare that they have no conflicts of interest.

## Supplementary Material

RA-015-D5RA05044H-s001

## Data Availability

The corresponding author will provide access to all the data used to support this research upon receiving an adequate request. Supplementary information includes Text S1: the sourcing and purity information of the used chemicals in this study; Text S2: the detailed information including instruments and methods used in catalyst characterization; Text S3: the detailed experimental procedure for the recycle study; Text S4: the analytical methods; Text S5: the details of operational approach of electrochemical impedance spectroscopy (EIS) and open-circuit potential (OCP); Fig. S1: synthesis schematic depiction; Fig. S2: the adsorption of acetaminophen by different catalysts; Fig. S3: the XPS survey spectra; Fig. S4: toxicity estimation. See DOI: https://doi.org/10.1039/d5ra05044h.
