# Supplementary material for: Rare earth doping: a strategy to enhance the catalytic activity of ZnFe2O4 in activating peroxymonosulfate for acetaminophen degradation
Source: RSC Adv. 2025 Aug 12;15(35):28651–8. doi: 10.1039/d5ra05044h (PMC12377269; doi:10.1039/d5ra05044h)
Supplement: RA-015-D5RA05044H-s001 [file RA-015-D5RA05044H-s001.pdf]

Supplementary Information (SI) for RSC Advances.
This journal is © The Royal Society of Chemistry 2025

Supplementary Information

Rare earth doping: A strategy to enhance the catalytic activity of $ZnFe_2O_4$ in activating peroxymonosulfate for acetaminophen degradation

Hangdao Qin [a,*], Lei Xiao, [a] Junnan Hao [a], Yong Wang [a], Jiming Huang [a], Guo Yang [b], Bo Xing [b]

[a] School of Material and Chemical Engineering, Tongren University, Tongren 554300, China

[b] College of Chemical Engineering, Sichuan University of Science and Engineering, Zigong 643000, China

* Corresponding author: Hangdao Qin

E-mail address: qinhangdao@126.com

Text S1: Acetaminophen was obtained from Shanghai Yien Chemical Technology Co. Ltd., China. $Zn(NO_3)_2·9H_2O$, $Fe(NO_3)_3·9H_2O$, $La(NO_3)_3·6H_2O$, $Pr(NO_3)_3·5H_2O$, Na_2SO_4, citric acid, NaOH, HNO_3, methanol (EtOH), *tert*-butanol (TBA), $NaHCO_3$, NaCl, $NaNO_3$, NaH_2PO_4, humic acid (HA) and 5,5-dimethyl-1-pyrroline-N-oxide (DMPO) were purchased from Sinopharm Chemical Reagent Co. Ltd., China.

Text S2: The crystallographic structure of the catalysts was characterized by X-ray diffraction (XRD) in the 2θ range of 10°-80° on a Rigaku Ultima IV diffractometer. The specific surface area, pore volume and average pore width of the catalysts were analyzed by N_2 adsorption-desorption automatic specific surface area analyzer (ASAP 2460, Micromeritics, USA). Analysis of surface chemical information for the catalysts was performed by X-ray photoelectron spectrometry (XPS, Thermo Scientific ESCALAB 250Xi, USA). The formation of the radicals in the reaction was determined by electron paramagnetic resonance spectroscopy (EPR, Bruker EMXPlus, Germany) using 5,5-dimethyl-1-pyrroline N-oxide (DMPO) as the trapping reagent.

Text S3: The reusability of $ZnLa_{0.5}Fe_{1.5}O_4$ catalyst was evaluated in four consecutive cycles. All the cycles were carried out under the same experiment conditions. At the end of each experiment, the solid catalyst was separated from water by centrifugal filtration, washed with ethanol and deionized water, and then dried in an drying oven at 60 °C overnight. The centrifugal filtration was performed at 2000 rpm for 5 min at the room temperature. No regeneration method was carried out in the first three cycles, and the used $ZnLa_{0.5}Fe_{1.5}O_4$ was heat-treated 2 h at 500 °C before the fourth cycle.

Text S4: Concentration of acetaminophen was analyzed quantitatively using the high performance liquid chromatography (HPLC, LC-20AD XR, Shimadzu) equipped with C18 column (4.6 × 250 mm, 5μm) and PDA detector at detection. The mobile phase was a mixture of acetonitrile and deionized water at a ratio of 50: 50 (v/v) with a flow rate of 0.8 mL/min. The detector wavelength was 243 nm and the injection volume was 20 μL.

Text S5: Firstly, 5 mg of solid catalyst was dispersed into 10 mL of 0.1 M acetic acid and 12 mg chitosan solution for 3 h ultrasonic treatment. In which, chitosan served as film-forming agent. Then, certain volume of the mixture was added to glassy carbon electrode until natural drying. The obtained catalyst coated electrode was used as working electrode for the subsequent electrochemical testing. Pt electrode and Ag/AgCl electrode (SCE) served as counter and reference electrode, respectively. EIS were measured with the initial potential of 0.2 V (amplitude of 0.0005 V) and frequency ranging from 0.01 to 100000 Hz, in 5 mM Na_2SO_4 solution with 0.1 M KCl. OPC test was conducted with the potential ranging from 0 to 2.0 V vs SCE with the scan rate of 0.02 V/s and the sensitivity of 10^{-4} (A/V). The electrolyte was 30 mL 20 mM Na_2SO_4 solution with/without the addition of 0.1g/L PMS and/or 10 mg/L acetaminophen.

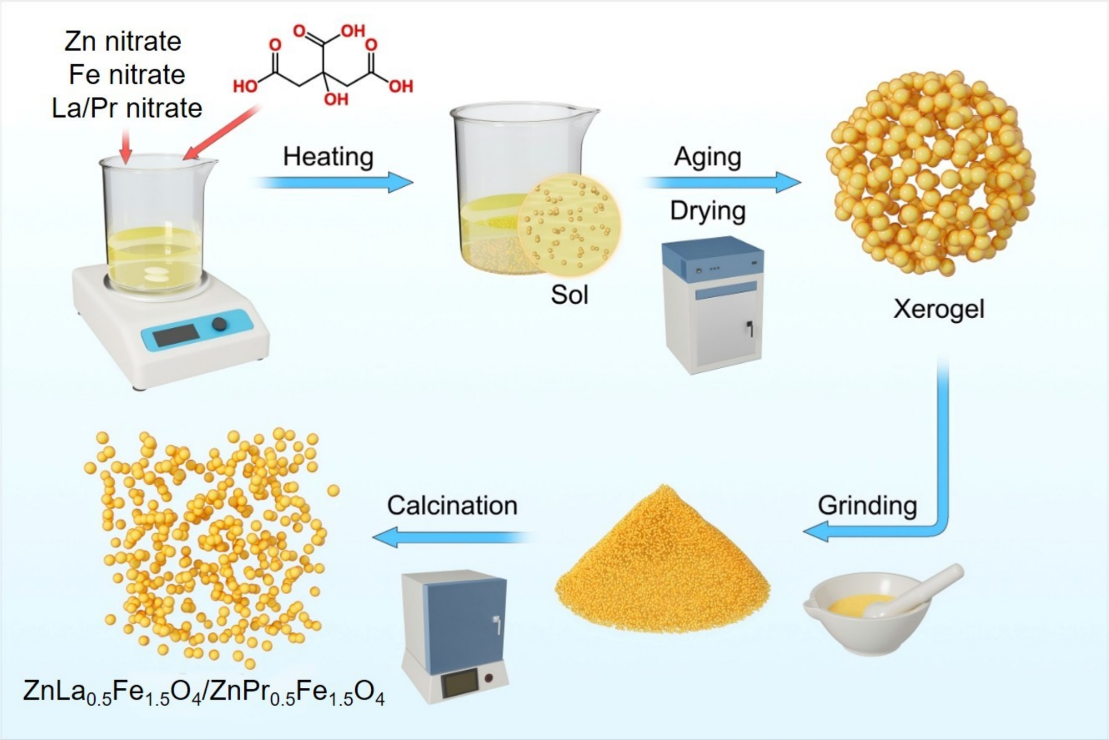

Fig. S1 Synthesis schematic depiction of $ZnLa_{0.5}Fe_{1.5}O_4$/$ZnPr_{0.5}Fe_{1.5}O_4$ catalyst.

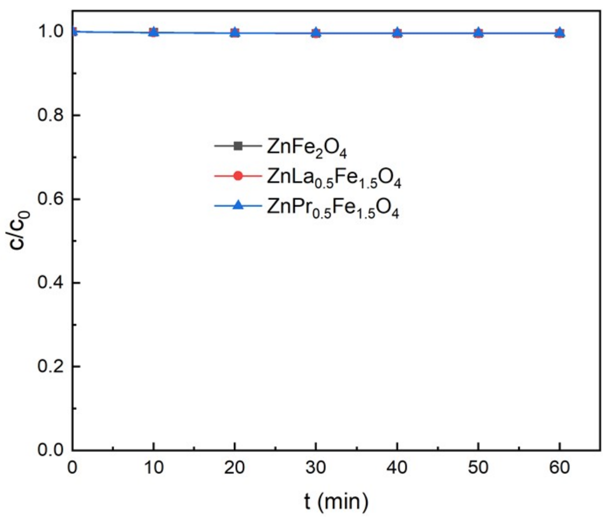

Fig. S2 The adsorption of acetaminophen by different catalysts.

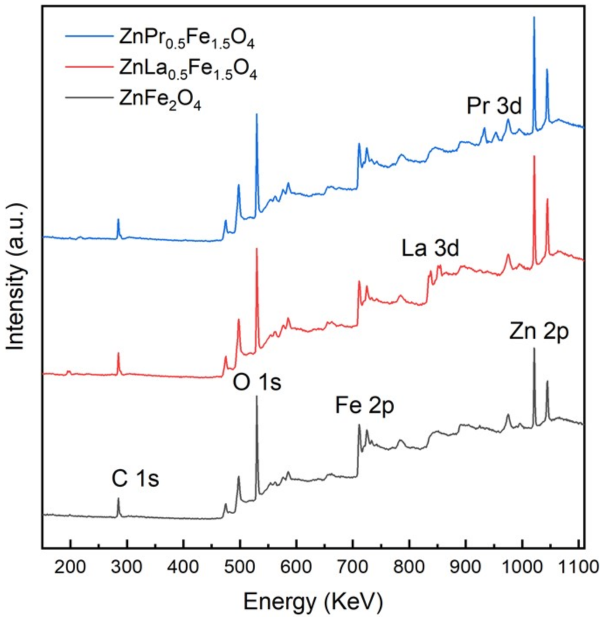

Fig. S3 The XPS survey spectra of $ZnFe_2O_4$, $ZnLa_{0.5}Fe_{1.5}O_4$ and $ZnPr_{0.5}Fe_{1.5}O_4$ catalysts.

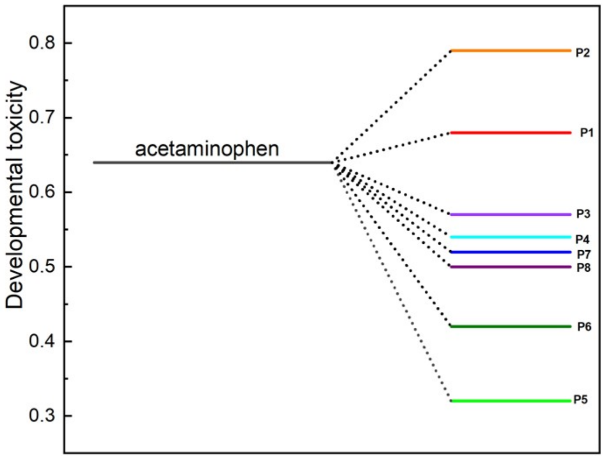

Fig. S4 Developmental toxicity of acetaminophen and its intermediates.
